# Relevance of Splicing on Tumor-Released Exosome Landscape: Implications in Cancer Therapeutics

**DOI:** 10.3389/fendo.2014.00194

**Published:** 2014-11-12

**Authors:** Elisa Oltra

**Affiliations:** ^1^Facultad de Medicina, Universidad Católica de Valencia “San Vicente Mártir”, Valencia, Spain

**Keywords:** tumor-released exosomes, dexosomes, splicing, cancer therapy, phage display, SELEX

Apart from apoptotic bodies and ectosomes, microvesicles that bud out of the plasma membrane, most types of cells release exosomes when intracellular endosomal microvesicular bodies fuse with their plasma membrane. This last type of vesicles with attributed intercellular communicating capabilities has acquired an enormous attention in the past two decades, especially for their potential as disease biomarkers and/or their use as therapeutic vehicles. The intense study of their molecular composition with special attention to miRNA has led to the development of databases like Exocarta (http://www.exocarta.org/) (1); however, the way exosomes released from tumors (Tex) that may influence cancer patient’s health is not yet completely understood.

The observation that exosome production and release is notably increased with tumor progression suggests that they must play an active role in cancer (2, 3). As tumors become more aggressive, heparanase activity is enhanced at least in myeloma, lymphomas, or breast cancer, increasing both the number of exosomes released and the amount of syndecan-1, VEGF, and HGF molecules exposed on their surface (4).

The advantages that Tex present for the delivery of tumorigenic signaling molecules in relation to passive release into the medium are clear, especially for transports across long distances in the body. Firstly, encapsulation of labile molecules such as RNAs and proteins within lipid bilayers offers them protection to degradation; secondly, the surface landscape of the vesicle allows for the possibility of tissue or cell specific targeting and thirdly they are suited for simultaneous multiple message delivery allowing horizontal transfer of complex information from cancer to healthy cells.

In addition to attenuation of antitumor immunity, Tex stimulate angiogenesis, modulate stromal cells activity, and help on the extracellular matrix remodeling contributing to the establishment of a premetastatic niche and generating suitable microenvironments at distant metastatic sites (5). For example, Tex from glioblastoma have the ability to potentiate tumor growth (6) and Tex from melanoma can directly tune a remote lymph node into a microenvironment that facilitates melanoma growth and metastasis even in the local absence of tumor cells (7). These and other similar studies suggest that Tex activities are mediated, at least in part, through the action of particular miRNAs, which presumably down regulate their target transcripts in recipient cells (8, 9). Interestingly enough, Fabbri et al. have recently showed that the Tex miR-21 and 29a molecules can bind to toll-like receptor 7 and 8 (TLR7 and 8) on immune cells and activate them, leading to TLR-mediated NF-kB activation and secretion of prometastatic inflammatory cytokines (10, 11). This offers an alternative mode of action for the miR-mediated Tex paracrine effects in which miR act as aptamer-ligands. So, in addition to Tex internalization Tex protein, lipid, carbohydrate, or nucleic acid surface receptors could interact with target cell receptors to activate intracellular signaling; or, their surface proteins could be cleaved by proteases, and the corresponding soluble fragments act as soluble ligands binding to target cell surface receptors (12). In one case or another, Tex-mediated activities are a threat to patients working toward disease progression. Importantly, anti-cancer agents directed to inhibit DNA replication or microtubule dynamics will result innocuous to Tex. This is, Tex present themselves, together with cancer stem cells, as a therapeutic resistant reservoir for the advancement of the disease, and therefore effective cancer treatments must include targeted inactivation and/or removal of Tex.

On another side, however, exosomes have emerged as potent stimulators of immune responses and from this point of view as agents for cancer therapy. It has been recently showed that induction of HSPs (heat shock proteins)-loaded Tex occurred when hepatocarcinoma cells were treated with chemotherapeutics such as paclitaxel or carboplatin. These Tex released by treated cancer cells conferred superior immunogenicity in inducing HSPs-specific NK cell responses (13). Exosomes can carry a broad variety of immune-stimulatory molecules depending on the cell of origin and *in vitro* culture conditions. In particular, dendritic cell (DC)-derived exosomes (dexosomes) have been shown to carry NK cell activating ligands and can be loaded with antigens to activate invariant NKT cells to induce antigen-specific T and B cell responses. Tumor-antigen-derived dexosomes have been investigated as therapeutic agents against cancer in two phase I clinical trials, with a phase II clinical trial currently ongoing. The results show that although dexosomes were well tolerated, the therapeutic success and immune activation were limited (14, 15). Multiple factors need to be considered in order to improve exosomal immunogenicity for cancer immunotherapy. For example, Tex immunostimulatory effect has been shown to depend on host’s DCs while dexosomes do not (16). Because of their abundant expression of tumor antigens, Tex can be envisaged as an acellular source of antigenic determinants to be exploited in the production of cancer-vaccines and therefore as a therapeutic cancer agent by itself or as a co-adjuvant treatment (17).

Although immune cells, and probably dexosomes, can be found in primary tumor lesions as infiltrating components playing favorable prognostic role (18), in metastatic lesions anti-cancer activities are suppressed due to Tex derived immunosuppressive effects allowing tumor progression. One of the mechanisms responsible for this undesired effect in different types of solid cancers uses endocytic FasL and also TRAIL loaded pro-apoptotic Tex to eliminate overreactive Fas-expressing T cells (19). Thus, Tex in malignant ascites effusions and other cancer patient fluids can eliminate activated T cells through a simple ligand–receptor interaction. In addition, functional CD39 and CD73 Tex are capable of dephosphorylating ATP and AMP to form adenosine to negatively regulate local immune responses (20). Tex can also inhibit DC differentiation, inhibit T-cell proliferation through TGF-β interactions, and promote tumor-immune evasion by interfering with NK cells (19) eventually hijacking the anti-cancer immune response that they might have initiated.

Reached this point, the key question for cancer therapeutics is: will patients benefit by the presence of Tex or effective removal of Tex should be recommended?

The answer does not seem to be straight forward but recent modeling for putative therapeutic implications of exosome exchange between tumor and immune cells may throw some light on it (21). The authors propose the existence of three cancer states: a low cancer load (L) with intermediate immune-level state, and intermediate cancer load (I) with high immune-level state and a high cancer load (H) with low immune-level state. To design and assess possible therapeutic protocols, the authors built a cancer-immunity landscape that includes dynamical states of the cancer-immunity interplay allowing for the visualization of combined protocols as trajectories connecting different states. For example, when the immune recognition is low, the effect of the immune system will not be strong enough to limit cancer progression.

An important observation that should be included in Lu et al. model is that exosome release is controlled by feedback regulatory loops that involve not only cancer cells but also exosome production by normal cells. In this sense, it has been reported that exosome release from normal human mammary epithelial cells and breast cancer cells is not only regulated by the presence of exosomes derived from their own cells but also exosomes from normal mammary epithelial cells inhibit exosome secretion by breast cancer cells in a tissue specific manner, implicating a dynamic equilibrium between exosome release of normal and tumor cells (22).

It is of relevance as well to point out that the different cancer-load-states may refer not only to quantitative traits but also to qualitative cancer-associated features. For example, cancer-specific membrane bound isoforms such as EGFRvIII, generated by defective alternative splicing mechanisms, have been detected on the surface of Tex (6). Such distinctive cancer-associated exons could be used for the design of directional therapeutics, allowing selective inactivation or sequestration of Tex with minimal interference of normal cell exosome activity, reducing possible secondary effects. In addition to antibody-based strategies, synthetic peptides and nucleic acid aptamers have proven not only to bind specifically and tightly to cancer cells but also to home to cancer cells *in vivo* (23–25). Similar approaches could be used to block or sequester Tex through their particular surface landscapes. Targeting through common receptors such as tetraspanins could interfere with normal exosome functions and therefore is not recommended.

The fact that solid tumors are heterogeneous in nature adds yet another degree of complexity to cancer therapeutics. Tumor heterogeneity will be reflected into a multivariety of Tex. Taking into account that the current methods for Tex isolation are based on vesicle size, density, and enrichment of certain common markers (tetraspanins) (26), we can conclude that Tex fractions are far from pure. A possible strategy to study more homogeneous Tex populations would be to establish cell lines from microdissected biopsies so that enough Tex of a particular type can be produced and isolated. This should allow for a better characterization of the Tex complex landscapes, some of them dependent on cancer-associated splicing switches.

Deregulation of splicing is associated to acquisition of cancer advantages, aggressiveness of the tumor, and drug resistance events (27–29). For example, the inclusion of an exon in the pyruvate kinase transcript allows for activation of glycolytic pathway of cancer cells (Warburg’s effect), and the KLF6-SV1 variant is associated with poor prognosis and tumor aggressiveness in prostate, lung, and ovarian cancers, associated to EMT (epithelial to mesenchymal transition) (30). In the context of directional targeting of Tex, it would be important to identify specific cancer-associated exons located on the external side of the vesicles. They may or may not be present in the plasmatic membrane of the tumor cell of origin. Toward this end, strategies such as screening of random libraries based on phage display and SELEX (Systematic Evolution of Ligands by EXponential enrichment) technologies allow for the identification of synthetic peptides and aptamers without previous knowledge of the altered splicing events (31, 32). Perhaps, tumor therapies suppressing the splicing machinery itself are advantageous to current systemic cancer treatments.

Tex cancer-associated exon sequences could be used for immunotherapy, to boost DC dexosome release and, at the same time, as targets for Tex inactivation and/or sequestration. If only inactivation/sequestration is applied, the patient would miss the benefit of the immune response against the tumor. On the other side, if the unloading of Tex is not prevented, their cargo will work toward advancement of the disease. This double-approach consisting on blocking Tex function while using their specific cancer-associated exon sequences to induce tumor-immune response could complement current tumor treatments to reduce relapses and to limit metastatic events in immuno-competent patients. In addition, this directional targeting strategy would leave exosome-dependent physiological functions unaffected.

In summary, the study of Tex surface landscape and the detection of cancer-specific exons on Tex derived from alternative splicing events results essential for the development of Tex targeted therapies. These qualitative switches should be considered in the context of patient immunological and cancer load states for the modeling and the development of improved, personalized cancer therapeutic programs.

## Conflict of Interest Statement

The author declares that the research was conducted in the absence of any commercial or financial relationships that could be construed as a potential conflict of interest.
